# Induction Therapy and Stem Cell Mobilization in Patients with Newly Diagnosed Multiple Myeloma

**DOI:** 10.1155/2012/607260

**Published:** 2012-05-31

**Authors:** Roberto Ria, Antonia Reale, Antonio Giovanni Solimando, Giuseppe Mangialardi, Michele Moschetta, Lucia Gelao, Giuseppe Iodice, Angelo Vacca

**Affiliations:** Section of Internal Medicine and Clinical Oncology, Department of Biomedical Sciences and Human Oncology, University of Bari Medical School, Policlinico, Piazza Giulio Cesare 11, I-70124 Bari, Italy

## Abstract

*Autologous stem cell transplantation* (ASCT) is considered the standard therapy for younger patients with newly diagnosed symptomatic *multiple myeloma* (MM). The introduction into clinical practice of novel agents, such as the proteasome inhibitor bortezomib and the *immunomodulatory derivatives* (IMiDs) thalidomide and lenalidomide, has significantly contributed to major advances in MM therapy and prognosis. These novel agents are incorporated into induction regimens to enhance the depth of response before ASCT and further improve post-ASCT outcomes. Between January 2000 and November 2011, 65 patients with MM were transplanted in the Department of Biomedical Science and Clinical Oncology at the University of Bari. According to Durie-Salmon, 60 patients had stage III of disease and 5 stage II. Only 7 patients were in stage B (renal failure). Induction regimens that were administered in two or more cycles were VAD (vincristine, adriamycin, and dexamethasone), Thal-Dex (thalidomide, dexamethasone), Len-Dex (lenalidomide, dexamethasone), Vel-Dex (bortezomib, dexamethasone), VTD (bortezomib, thalidomide, and dexamethasone), and PAD (bortezomib, pegylated liposomal doxorubicin, and dexamethasone). In mobilization procedure, the patients received cyclophosphamide and *granulocyte colony-stimulating factor* (G-CSF). The number of cells collected through two or more leukapheresess, response after induction, and toxicity were evaluated to define the more adequate up-front induction regimen in transplantation-eligible MM patients.

## 1. Introduction

Over the last decade, high-dose chemotherapy followed by ASCT has been considered the standard of care for younger patients with newly diagnosed MM, based on the increased rate of *complete response* (CR), prolonged* overall survival* (OS), and reduced side effects compared with conventional chemotherapy in several randomized studies [1–4]. Patients who are eligible for early ASCT typically receive a limited number of cycles of induction therapy to reduce tumor cell mass and bone marrow plasma cell infiltration before collection of peripheral blood stem cells [5]. Compared with conventional treatments used in the past, a number of novel agents are now available which affect increased rates of CR [6–8]. The reach of CR after both induction therapy and ASCT is one of the strongest predictors of long-term outcomes and represents a major endpoint of the treatment strategy in younger patients together with maintenance of a durable CR [5].

The initial rationale for the use of IMiDs in MM was their known antiangiogenic activity, considered the primary role of increased angiogenesis in MM bone marrow [9, 10]. Further investigations have shown that besides their direct effect on MM *plasma cells* (PCs) thalidomide and lenalidomide, they abrogate the adhesion of MM cells to the bone marrow stromal cells and block the secretion of growth factors, survival factors, angiogenic cytokines (*interleukin *(IL)-6, *tumor necrosis factor* (TNF)-alpha, *vascular endothelial growth factor* (VEGF), and *fibroblast growth factor* (FGF)-2) triggered by the cross talk between tumor cells and their microenvironment [11, 12]. Additionally these agents significantly modulate the host immune response by expanding the number and the function of *natural killer* (NK) cells, improving the *dendritic cell* (DC) functions, and enhancing T-cell functions by providing T-cell costimulatory signals through B7/CD28 pathways [13].

Bortezomib is the first proteasome inhibitor used in clinical practice in MM since the discovery of its antitumoral mechanisms: the blockade of NF-*κ*B activation and the IL-6 paracrine loop [14, 15]. Bortezomib has been subsequently demonstrated to act directly on PCs to induce apoptosis through both caspases 8 and 9 activation, and it overcomes the protective effect of IL-6, and it acts synergistically with Dexamethasone [16]. Similarly to IMiDs, bortezomib explicates its activity on the bone marrow microenvironment inhibiting the PCs binding to stromal cells, the secretion of growth factors, and myeloma-associated neoangiogenesis [16].

The goal of CD34+ cell mobilization is to collect enough cells to achieve a rapid and sustained hematopoietic recovery after high-dose therapy, since delayed hematopoietic recovery correlates with increased toxicity and transplant-related mortality. It has been demonstrated that high CD34+ cell doses (>3/5 × 10^6^/kg) are associated with faster hematological recovery and lower incidence of infectious and bleeding complications [17]. Doses <2 × 10^6^/kg are associated with slower recovery and worse outcomes. CD34+ cell doses over 15 × 10^6^/kg after high-dose melphalan administration can eliminate severe thrombocytopenia. The International Myeloma Working Group (IMWG) has suggested a minimum target of 4 × 10^6^ and, if feasible, an average of 8–10 × 10^6^/kg that should be collected, allowing most myeloma patients to undergo two autografts during the course of their disease, also considering that in some patients, the first ASCT can be unsuccessful [18].

We also evaluated the drug-related toxicity. Concerning IMiDs, common side effects are deep vein thrombosis [19, 20] and related pulmonary embolism, neutropenia and thrombocytopenia, peripheral neuropathy, fatigue, and constipation. Bortezomib toxicity instead is mainly due to peripheral neuropathy and thrombocytopenia [21]. Diarrhea and fatigue are also observed. VAD increased mortality mainly due to systemic infections [22].

## 2. Materials and Methods

### 2.1. Patients

In this study, a total of 65 patients (44 men and 21 women), aged 48–65 years (median age 56.5 years), with symptomatic MM (Durie-Salmon stage II/III) have been evaluated (Table 1).

Data from all adult patients with MM (*n* = 65) scheduled for stem cell collection and subsequent ASCT between 2000 and 2011 at the Department of Biomedical Science and Clinical Oncology, Section of Internal Medicine and Human Oncology at the University of Bari, Italy, were analyzed in this retrospective study.

This study was approved by the Local Ethics Committee, and all patients gave their informed consent according with the Declaration of Helsinki.

### 2.2. Treatments

Induction therapy consisted of bortezomib-based regimens (VTD [23], Vel-Dex [24], and PAD [25]); Lenalidomide-based regimens (Len-Dex [26]); other regimens (VAD [22], Thal-Dex [27, 28]).

VAD consisted of two 28-day cycles. Vincristine was administered intravenously (i.v.) at the dose of 1 mg/m^2^ on day 1; adriamycin 9 mg/m^2^ on days 1–4; dexamethasone 40 mg daily on days 1–4.

VTD comprised four 3-week cycles of Bortezomib 1,3 mg/m^2^ on days 1, 4, 8, and 11, thalidomide 100 mg/day for the first 14 days and 200 mg daily thereafter administered orally, and dexamethasone at the same dose and schedule as for the Vel-Dex regimen. Recommended concomitant medications included bisphosphonates, antibiotics, and antiviral and antithrombotic prophylaxis in accordance with local practice.

Thal-Dex was administered as four 35-day cycles, thalidomide at the dose of 100 mg/day for 7 days orally, and dexamethasone 40 mg orally or i.v. on days 1–4, 9–12, and 17–20.

Len-Dex consisted of four 28-day cycles. Lenalidomide was administered orally at the dose of 25 mg on days 1–21 and dexamethasone orally at the dose of 40 mg on days 1–8–15–22.

Vel-Dex consisted of four 3-week cycles of bortezomib 1.3 mg/m^2^ administered i.v. on days 1, 4, 8, and 11, plus dexamethasone 40 mg days 1–4 (all cycles) and 9–12 (cycles 1 and 2).

PAD was administered as four 21-day cycles comprising bortezomib 1,3 mg/m^2^ i.v. on days 1, 4, 8, and 11, doxorubicin at 30 mg/m^2^ i.v. on days 1 to 4, and dexamethasone 40 mg p.o., on days 1 to 4.

After induction therapy, all patients received a single infusion of cyclophosphamide 4 g/m^2^ followed by daily administration of subcutaneous G-CSF at the dose of 10 *μ*g/kg from day +5 until the peripheral stem cell harvest [29]. Nineteen patients received a second infusion of cyclophosphamide; of these, 8 patients received plerixafor as an additional mobilizing agent [30].

### 2.3. Response Criteria

Response, relapse, and progression were assessed according to the *European Bone Marrow Transplant* (EBMT) criteria [31]. CR was defined as negative serum and urine immunofixation, <5% plasma cells in a bone marrow aspirate as well as disappearance of soft tissue plasmacytomas and no increase in lytic bone lesions. *Partial response* (PR) was defined as a decrease of serum M-protein in ≥50%, 24-hour urinary light-chain excretion by ≥90% or to <200 mg, and reduction of extramedullary plasmacytomas in ≥50%. *Minimal response* (MR) required a reduction in the level of serum protein between 25–49% and 50–89% reduction in 24 hours urine light-chain protein excretion. Responses should be maintained for a minimum of 6 weeks. Relapse from CR was defined as reappearance of serum or urinary paraprotein on immunofixation, development of new extramedullary plasmacytomas, increase in size, or developing of new lytic lesions or hypercalcemia. Progressive disease required an increase in serum M-protein by >25% with an absolute increase of at least 5 g/L or increase in urine M-protein by >25% and also an absolute increase ≥200 mg/24 h, and/or the appearance of soft-tissue plasmacytomas, new lytic bone lesions, or hypercalcemia.

### 2.4. Toxicity

All the adverse events were considered during the induction and mobilization period according to the *National Cancer Institute Common Toxicity Criteria* (version 3.0).

### 2.5. Statistical Analysis

Data were analyzed with the SPSS (Chicago, IL, USA) software package. All results are presented as median ±1 SD and range. The medians were compared with the Mann-Whitney *U* test. *P* values < 0.005 were considered significant. One-way ANOVA analysis was used to compare all parameters between patients treated with bortezomib or lenalidomide or other agents, also with respect to response, number of collected cells, and toxicity.

## 3. Results and Discussion

### 3.1. Results of Mobilization and Leukapheresis

**Figure 1:**
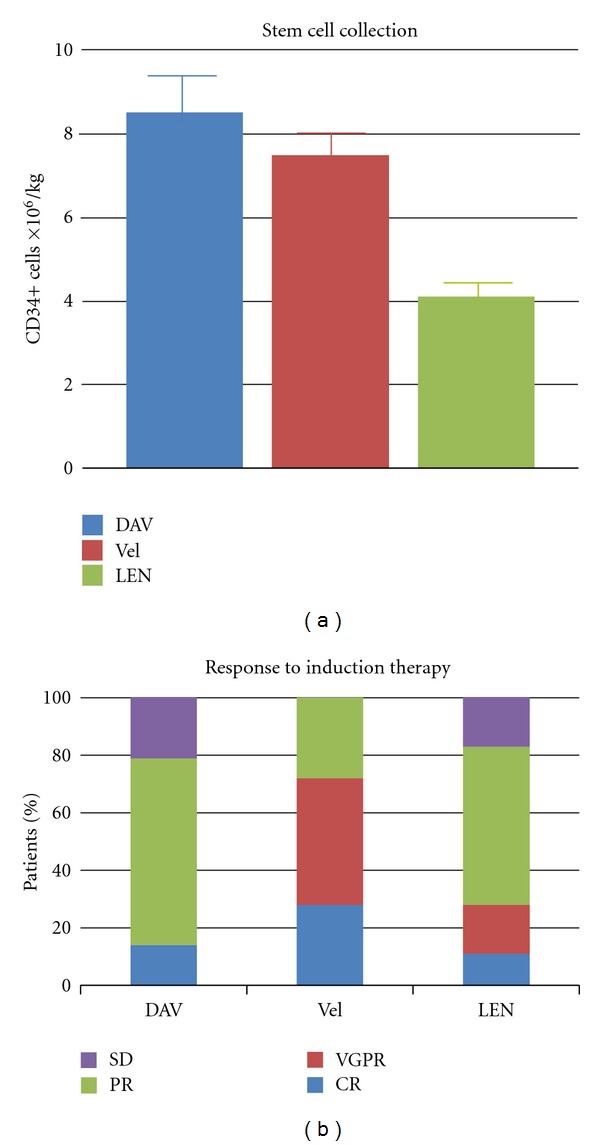


In VAD- and Vel-treated patients, a significantly higher CD34+ collection was obtained: 8.5 ± 1.01 × 10^6^ CD34+/Kg body weight (bw) and 7.5 ± 1.08 × 10^6^ CD34+/Kg bw, respectively, versus 4.11 ± 0.60 × 10^6^ CD34+/Kg bw in lenalidomide-treated patients (*P* = 0.01 and *P* < 0.005) (Figure 1(a)). The percentage of patients who reached the minimum collection target after two leukaphereses was higher in those treated with VAD or Vel (86% and 84%, resp.) than those treated with lenalidomide (56%) (*P* < 0.01). The apheresis' mean volume after plasma detraction was of 56 ± 16 mL in VAD- and Vel-treated patients and 53 ± 13 mL in lenalidomide treated patients.

### 3.2. Efficacy

*Overall response rate* (ORR) was significantly better in Vel-based regimens (100% versus VAD 79% and versus lenalidomide 83%). Particularly in VAD-treated patients, we observe 4% of CR, 19% of PR, and 6% of *stable disease* (SD). In Vel regimen, we found 5% of CR, 8% of VGPR, and 5% of PR. Finally, response to lenalidomide was 2% of CR, 3% of *very good partial response* (VGPR), and 10% of PR; 3% patients showed SD.

### 3.3. Adverse Events

Hematologic and nonhematologic toxicities are listed in Table 2. No peripheral neuropathy was observed in all treatment regimens. Thrombocytopenia was the most frequent observed side effect and presented the highest incidence in both the novel agent groups, although in Len-treated patients, this was more serious (grade III/IV). Anemia and nausea were also frequently observed. In this retrospective analysis, the overall occurrence of adverse events during treatment was higher in Len-based regimens.

### 3.4. Discussion

Data from several clinical trials have demonstrated the superiority of new agents either in combination or not with conventional chemotherapy as up-front therapy for newly diagnosed MM young patients. Cavo et al. [32] showed the pivotal role of IMiDs as primary therapy in preparation for autologous transplantation compared with cytotoxic protocols. The introduction of bortezomib in the 2000s has dramatically changed the treatment options and the clinical outcomes of both relapsed/refractory [33] and newly diagnosed myeloma patients [24]. Furthermore, it has been extensively clarified that doublet therapies combining either an IMiD or bortezomib with dexamethasone (i.e., Thal-Dex or Len-Dex or Vel-Dex) affected higher overall response rates, *progression free survival* (PFS), and less toxicity than traditional treatments [34–39]. Nevertheless, the Italian group [23] in a randomized phase III study has demonstrated that the combination of Vel-Thal-Dex (VTD) improves the rate of CR and near-CR with no increase of relevant toxicity. So, this combination can actually be considered the gold standard for induction therapy in younger transplant-eligible patients.

Lenalidomide is a more active analogue of thalidomide. This provides the basis for its role in newly diagnosed MM patients. When combined with dexamethasone and used beyond four cycles, it achieves over 90% PR or better rate [39]. A similar clinical trial in Europe had almost identical results [26]. On the other hand, myelosuppression induced by lenalidomide represents the dose-limiting toxicity and requires monitoring during therapy. Similarly to thalidomide, lenalidomide is associated with increased incidence of deep vein thrombosis and requires concurrent prophylactic measures for its prevention [40, 41].

According to our data, the use of Vel-Dex regimen in newly diagnosed patients has a strong impact in terms of CR/nCR, VGPR, and ORR. Jagannath et al. [24] reported 18% CR and 88% ORR in a phase II study using combination of Vel-Dex in newly diagnosed MM patients. The *International Francophone Group against Myeloma* (IFM) has randomized 242 newly diagnosed patients to Vel-Dex or VAD: CR/nCR were 15% versus 6% at least, VGPR 38% versus 15%, and ORR 79% versus 63% after four cycles of induction therapy. They observed significantly higher ORR with Vel-Dex compared with VAD [36].

In line with our observations, prolonged lenalidomide induction therapy has been reported to affect stem cell mobilization. Patients undergoing peripheral blood stem cell mobilization with G-CSF following lenalidomide induction had significant decrease in total CD34(+) cells collected, average daily collection, and increased number of aphereses [42]. The exact mechanisms by which lenalidomide interferes with stem cell mobilization are not clear. However, it seems that there are no harmful effects on the quality of PBSC collected as denoted by similar engraftment rate across all treatment groups. Nevertheless, here we observed that the frequency and the seriousness of lenalidomide-induced adverse events are higher compared to those observed in the Vel or other therapy-based regimens. Sekeres et al. [40] estimated that more than half of patients treated with lenalidomide-based protocols developed grades 3 and 4 cytopenia, mostly neutropenia and thrombocytopenia. Interestingly, it has been shown that patients who develop severe hematologic toxicity are more likely to better mobilize after cyclophosphamide therapy, but mechanisms beyond this clinical evidence remain still obscure. Contrarily, we did not observe any Vel-related severe adverse events delaying ASCT. Based on these reports, no more than six months of Lenalidomide including regimen prior to Cyclophosphamide mobilization should be recommended to avoid poor PBSC collection [43]. However, the recent introduction of plerixafor which increases the number of mobilized circulating hematopoietic stem and progenitor cells when administered with G-CSF may improve PBSC collection and change this scenario.

## 4. Conclusion

In the present study, we confirm that to date a precise up-front induction regimen for ASCT cannot be strongly advised; each induction regimen presents some benefits and disadvantages that should be considered on the basis of medical history and comorbidities of single myeloma patients. However, novel antimyeloma drugs, mostly bortezomib- and lenalidomide-based regimens, offer a higher percentage of ORR, and CR/nCR rate compared to other treatments based on conventional agents. Notably, attainment of CR after both induction therapy and ASCT is one of the strongest predictors of long-term outcomes and represents a major endpoint of current treatment strategies incorporating autotransplantation upfront. Among novel agents, bortezomib containing associations offer a better ORR, toxicity profile, and a higher probability to collect the minimum target of CD34+.

## Figures and Tables

**Table 1:** Patients' characteristics.

Characteristics	Total number of patients	Treated with bortezomib	Treated with lenalidomide	Treated with other agents
Numbers	65	18	18	29
Sex, M/F	44/21	10/8	11/7	23/6
Age				
Median (range) years	56.5 (48–65)	56 (48–64)	58 (51–65)	57.5 (50–65)
Monoclonal component				
IgG/IgA/*κ*/*λ*	37/19/6/3	9/5/1/3	10/6/2/0	18/8/3/0
Durie-Salmon stage				
I/II/III	0/5/60	0/0/18	0/2/16	0/3/26
A/B	58/7	14/4	17/1	27/2
ISS stage				
1/2/3	20/27/18	6/7/5	8/6/4	6/14/9
Mobilizing chemotherapy				
one/two	46/19	13/5	10/8	23/6
Number of leukapheresis				
Two/three	31/34	18/0	2/16	11/18

**Table 2:** Adverse events.

	Total adverse events	Treated with bortezomib	Treated with lenalidomide	Treated with other agents
Fatigue	2	0	2	0
Deep vein thrombosis	1	0	1	0
Thrombocytopenia	13			
Grade I/II		5		2
Grade III/IV			6	
Anemia	11	0	9	2
Exacerbation of Crohn's disease	1	0	1	0
Mucositis grade II	3	2	1	0
Nausea grade II	11	2	8	1
Diarrhea grade I/II	3	2	1	0
Dermatological toxicity	3	3	0	0
